# Insights into the protein domains of C-VI TRIM subfamily in viral infection

**DOI:** 10.3389/fcimb.2025.1573422

**Published:** 2025-05-12

**Authors:** Bbumba Patrick, Yan-Chung Lo, Wen-Chi Su

**Affiliations:** ^1^ Graduate Institute of Biomedical Sciences, China Medical University, Taichung, Taiwan; ^2^ Sinphar Pharmaceutical Co., Ltd., Sinphar Group, Yilan, Taiwan; ^3^ International Master’s Program of Biomedical Sciences, China Medical University, Taichung, Taiwan; ^4^ Department of Medical Research, China Medical University Hospital, Taichung, Taiwan; ^5^ Drug Development Center, China Medical University, Taichung, Taiwan

**Keywords:** TRIM28, TRIM24/TRIM28/TRIM33 complex, RBCC domain, C-VI TRIM PROTEINS, TRIM24, Trim33, virus

## Abstract

Tripartite motif (TRIM) proteins, defined by their conserved RBCC domain architecture, play key roles in various cellular processes and virus-host interactions. In this review, we focus on Class VI TRIM proteins, including TRIM24, TRIM28, and TRIM33, highlighting the distinct functional attributes of their RING, B-BOX1, B-BOX2, COILED COIL, PHD, and BRD domains in viral infection. Through multiple sequence alignment, we delineate both the conserved and divergent features within this subclass, underscoring the uniqueness of Class VI TRIM protein. Additionally, we explore the post-translational modifications (PTMs) of Class VI TRIM proteins including their functional differences in modulating viral infection. Moreover, we examine the C-VI TRIM protein complexes and their significant contributions to the antiviral response. Furthermore, we discuss small molecule ligands targeting Class VI TRIM domains, with a focus on druggable structural motifs. Understanding the multi-domain features of TRIM proteins is crucial for developing effective antiviral strategies and the therapeutic modulation of their activity.

## 1 Introduction

**Figure 1:**
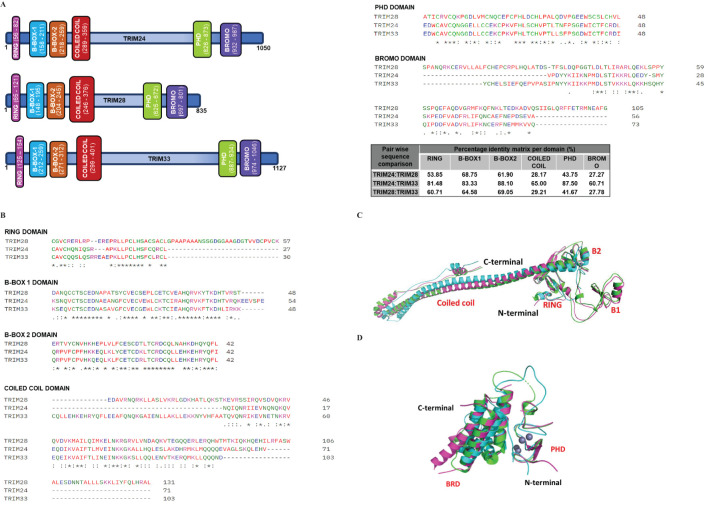
Schematic and 3D representation of Class VI TRIM proteins. **(A)** The schematic representation of Class VI TRIM proteins for TRIM24, TRIM28, and TRIM33, respectively. These proteins share a similar structural arrangement, indicative of conserved domain architecture. Among them, TRIM33 has the highest molecular mass (1127 kDa), followed by TRIM24 (1050 kDa) and TRIM28 (835 kDa). The domains are color-coded as follows: Magenta (RING), Blue (B-BOX1), Orange (B-BOX2), Red (COILED COIL), Green (PHD), and Purple (BROMO). **(B)** Multiple Sequence Alignment of Class VI TRIM domains, including RING, B-BOX1, B-BOX2, COILED COIL, PHD, and BROMO domains. The alignment was performed using CLUSTAL Omega software. Gaps in the sequences are indicated by dashes (—), with asterisks (*), colons (): and dots (.) representing identical residues, conserved residues, and semi-conserved residues, respectively. The percent identity matrix for each TRIM domain comparison is displayed in the table. The domains of TRIM24 and TRIM33 exhibit greater similarity to each other than to those of TRIM28. Nevertheless, all domains demonstrate high conservation. The UniProt accession numbers for TRIM24, TRIM28, and TRIM33 are O15164, Q13263, and Q9UPN9, respectively. **(C, D)** The 3D structures of C-VI RBCC **(C)** and PHD-BROMO **(D)** domains are superimposed depicting their similar structural orientation. The PDB accession numbers and colour codes are Green-TRIM24 (PDB code: AF-O15164-F1-model_V4 residuals 47-425), Cyan-TRIM28 (PDB code: 6QAJ residuals 56-405), Magenta-TRIM33 (PDB code: AF-Q9UPN9-F1-model_V4 residuals 116-478) **(C)**. Green-TRIM24 (PDB code: 3O34 residuals 823-1006), Cyan-TRIM28 (PDB code: 2RO1 residuals 627-755), and Magenta-TRIM33 (PDB code: 3UN5 residuals 881-1056) **(D)**.

Tripartite motif (TRIM) proteins are defined by a conserved N-terminal RBCC (RING finger, B-box, coiled-coil) domain and a variable C-terminal region, classified into 11 subfamilies (C-I to C-XI) based on their C-terminal domain compositions (Short and Cox, 2006; Ozato et al., 2008). The RBCC domain comprises a RING finger domain, one or two B-box domains, and a coiled-coil domain (Carthagena et al., 2009) (**Figure 1A**). The RING domain, a specialized zinc finger, confers E3 ubiquitin ligase activity, while B-box domains, also zinc-binding motifs, facilitate protein-protein interactions, though their precise roles remain unclear (Massiah, 2019). The coiled-coil domain mediates anti-parallel homo-dimerization and may enable hetero-oligomerization (Stevens et al., 2019). TRIM proteins are critical modulators of signaling pathways in development and tumorigenesis (Herquel et al., 2011) and play dual roles in promoting or inhibiting viral infections through diverse mechanisms (Vunjak and Versteeg, 2019).

Within the broad TRIM family, class VI (C-VI) TRIM proteins, which belong to TIF1 (transcriptional intermediary factor 1) family members, are particularly noteworthy for their complex domain architecture. The C-VI TRIM proteins include TRIM24 (TIF1α), TRIM28 (TIF1β, KAP1), and TRIM33 (TIF1γ). These proteins function as chromatin-associated transcriptional co-regulators, driven by their C-terminal PHD (plant homeodomain) and BRD (bromodomain) domains (Stevens et al., 2019) (**Figure 1A**). The PHD domain, a zinc finger structure, coordinates zinc ions in a cross-brace configuration, while BRD domains feature a conserved four-α-helix bundle with variable loop regions for motif recognition (Sanchez and Zhou, 2011).

C-VI TRIM proteins exhibit high domain conservation, with TRIM24 showing significant similarity to TRIM33 across domains (**Figure 1B**). Structural studies using NMR and X-ray crystallography have resolved key domains, including the TRIM28 RBCC (PDB 6QAJ) (Randolph et al., 2022), TRIM24 PHD-BROMO (PDB 4YAB) (Palmer et al., 2016), and TRIM33 PHD-BROMO (PDB 7ZDD) (Sekirnik et al., 2022). However, full-length structures remain elusive due to intrinsic disorder in the linker region (Fonti et al., 2019; Randolph et al., 2022). Recent AlphaFold models have provided insights into their full-length structures, revealing distinct domain orientations (Varadi et al., 2024) (**Figures 1C, D**). This review explores the unique roles of C-VI TRIM proteins, focusing on their shared domain compositions and domain functions during viral infection.

## 2 The uniqueness of class VI TRIM proteins

Class VI TRIM proteins are uniquely characterized by their C-terminal PHD-BRD motif, distinguishing them within the TRIM family (Randolph et al., 2022). These tandem domains enable recognition of specific histone modifications, such as methylated and acetylated lysine residues, allowing them to function as epigenetic readers (Sanchez and Zhou, 2011; Zaware and Zhou, 2019). TRIM24, for instance, interacts with histone tails and nuclear receptors via its LXXLL motif, regulating transcriptional programs critical for cell proliferation (Walser et al., 2016; Tsai et al., 2022). TRIM28 acts as a scaffold, recruiting repressive complexes through its PHD-BRD cassette and PxVxL motif, facilitating heterochromatin formation and gene silencing (Thiru et al., 2004; Mazurek et al., 2021). Similarly, TRIM33 binds H3K9me3 and K18ac, displacing HP1γ to enhance transcriptional activation (Xi et al., 2011). These proteins play pivotal roles in chromatin remodeling, transcriptional regulation, and antiviral responses (Rajsbaum et al., 2014).

## 3 The role of C-VI TRIM domains in viral infection

Class VI TRIM proteins, including TRIM24, TRIM28, and TRIM33, exhibit high conservation of amino acid residues across species, underscoring their structural and functional homology and their critical roles in cellular processes, including antiviral and proviral mechanisms (Shibata et al., 2011). While the broader roles of TRIM proteins in host-virus interactions, ubiquitin ligase activity, and antiviral innate immune signaling have been extensively reviewed (van Gent et al., 2018; Hage and Rajsbaum, 2019; Giraldo et al., 2020; Shen et al., 2021), the specific contributions of TRIM domains in viral infections remain less explored. This section highlights the role of Class VI TRIM protein domains, including the RBCC domain and individual structural domains, in viral pathogenesis and summarizes the findings in **Table 1**.

**Table 1:** The table summarizing the role of C-VI TRIM domains in viral infection.

Member of C-VI TRIM	Virus	Domain involved	Influence of the domain	Reference
TRIM28	Prototype foamy virus (PFV)	RBCC	It destabilizes Tas, reducing its activation of viral promoters and repressing PFV transcription and replication.	(Yuan et al., 2021)
TRIM28	Vesicular stomatitis virus (VSV) and Herpes simplex virus 1 (HSV-1)	RBCC	It mediates TBK1 K63-linked ubiquitination and subsequent IFN-I induction, thereby positively regulating the antiviral immune response.	(Hua et al., 2024)
TRIM33	Spring Vremia of Carp virus (SVCV)	RBCC	It degrades viperin_sv1, suppressing the type-1 interferon response and enhancing SVCV replication.	(Gao et al., 2021)
TRIM28	Porcine epidemic diarrhea virus (PEDV)	RING domain	It interacts with the PEDV N protein to induce mitophagy, which suppresses the JAK/STAT1 pathway and enhances viral replication.	(Li et al., 2024)
TRIM28	Sendai virus	RING domain	It inhibits SeV-induced RLR signaling by facilitating the polyubiquitination and proteasome-mediated degradation of MAVS.	(Chen et al., 2023)
TRIM28	Sendai virus	RING domain	It promotes the SUMOylation of IRF7, reducing its activity and subsequently decreasing IFN production during viral infections.	(Liang et al., 2011)
TRIM24	Vesicular stomatitis virus (VSV) and Herpes simplex virus 1 (HSV-1)	RING domain	It promotes TRAF3 ubiquitination, leading to its interaction with MAVS and TBK1, and activating antiviral signaling.	(Zhu et al., 2020)
TRIM33	HIV-1	RING domain	It drives poly-ubiquitination of HIV-1 integrase, marking it for proteasomal degradation and inhibiting proviral DNA formation and HIV-1 replication.	(Ali et al., 2019)
TRIM28	Porcine Reproductive and Respiratory Syndrome Virus (PRRSV)	BBOX + CC	It prevents PRRSV GP4 degradation, enhancing GP4 expression and supporting PRRSV replication.	(Cui et al., 2023)
TRIM28	SARS-CoV-2	Coiled coil	It facilitates the association of TRIM28 with SARS2-NP enabling the virus to evade the host’s innate immune response.	(Ren et al., 2024)
TRIM28	VSV	PHD	It suppresses antiviral gene transcription during VSV infection.	(Kuang et al., 2023)
TRIM28	HIV-1	BRD	It suppresses HIV-1 gene expression.	(Ait-Ammar et al., 2021)

### 3.1 The influence of the RBCC domain in virus infection

The RBCC domain comprising the RING, B-box, and coiled-coil regions, plays a versatile role in viral infections, either enhancing or inhibiting viral activity depending on the context. For instance, TRIM28 restricts prototype foamy virus (PFV) replication by promoting the ubiquitination and degradation of the viral transactivator Tas via its RBCC domain. This interaction suppresses PFV transcription and replication while maintaining repressive H3K9me3 marks at viral LTR promoter regions, facilitating viral latency (Yuan et al., 2021). Similarly, during infections with RNA (VSV) and DNA (HSV-1) viruses, the RBCC domain of TRIM28 is essential for binding TBK1 and facilitating its K63-linked ubiquitination, which is critical for type I interferon (IFN-I) activation. TRIM28 knockout cells exhibit impaired IFN-I responses and increased viral susceptibility, highlighting the RBCC domain’s importance in antiviral defense (Hua et al., 2024). Additionally, the RBCC domain of TRIM33 interacts with the antiviral protein viperin_sv1 during Spring viremia of carp virus (SVCV) infection, inducing its proteasomal degradation. This process dampens the type-1 interferon response, thereby enhancing SVCV replication (Gao et al., 2021).

### 3.2 The influence of the RING domain in virus infection

The RING domain of TRIM proteins plays a pivotal role in viral infections by mediating protein-protein interactions and facilitating ubiquitination and SUMOylation processes (McAvera and Crawford, 2020). In the context of porcine epidemic diarrhea virus (PEDV), an enteropathogenic coronavirus, the TRIM28 RING domain binds to the viral nucleocapsid protein, triggering mitophagy and suppressing the JAK/STAT1 signaling pathway, which is essential for antiviral defense. Depletion of TRIM28 restores JAK/STAT1 signaling and impairs PEDV replication, while its overexpression enhances viral replication, underscoring its role in viral exploitation of host mechanisms (Li et al., 2024). These findings suggest that targeting TRIM28 could be a viable therapeutic strategy against PEDV. Similarly, during Sendai virus (SeV) infection, TRIM28 suppresses RIG-I-like receptor (RLR) signaling by targeting MAVS for K48-linked polyubiquitination, a process dependent on specific cysteine residues in its RING domain. Additionally, TRIM28 acts as a SUMO E3 ligase for IRF7, enhancing its SUMOylation and negatively regulating type I interferon responses. Overexpression of TRIM28 inhibits IRF7 activity, while its knockdown enhances antiviral defenses, highlighting its dual role in immune regulation (Liang et al., 2011; Chen et al., 2023).

In Vesicular stomatitis virus (VSV) infection, the RING domain of TRIM24 is essential for its antiviral function. TRIM24 promotes K63-linked ubiquitination of TRAF3, facilitating its interaction with MAVS and TBK1 to activate antiviral signaling. The downregulation of TRIM24 by VSV-activated IRF3 compromises type I interferon induction, increasing host susceptibility to infection (Zhu et al., 2020). Furthermore, the RING domain of TRIM33 is critical for its role in restricting HIV-1 replication. TRIM33 catalyzes the polyubiquitination of HIV-1 integrase (IN), targeting it for proteasomal degradation and preventing proviral DNA formation. Mutations in the RING domain, but not the PHD domain, impair this function, emphasizing its importance as a cellular restriction factor (Ali et al., 2019). Collectively, these studies highlight the central role of TRIM RING domains in modulating antiviral immune responses and viral replication, offering potential targets for therapeutic intervention.

### 3.3 The influence of the BCC domain in virus infection

The B-box and coiled-coil (CC) domains of TRIM proteins are critical for the formation of higher-order assemblies and play significant roles in viral infections, including those caused by porcine reproductive and respiratory syndrome virus (PRRSV) and SARS-CoV-2. In PRRSV, the envelope glycoprotein 4 (GP4) is essential for producing infectious viral particles (Meulenberg et al., 1997). TRIM28, through its B-box and CC domains, enhances PRRSV GP4 expression by directly interacting with GP4, inhibiting its K63-linked ubiquitination, preventing its degradation, and stabilizing the protein to promote PRRSV replication. This domain-specific function highlights TRIM28 as a potential target for antiviral therapies against PRRSV (Cui et al., 2023). Similarly, in SARS-CoV-2 which causes COVID-19 disease (Wihandani et al., 2023), the CC domain of TRIM28 is crucial for viral virulence. It facilitates the interaction between TRIM28 and the SARS-CoV-2 nucleocapsid protein (SARS2-NP), enabling poly-SUMOylation of SARS2-NP, which helps the virus evade host innate immune responses. Depriving SARS2-NP of SUMOylation increases IFN-β expression, reduces viral propagation, and lowers mortality in mice (Ren et al., 2024). Additionally, the CC domain mediates interactions with the Krüppel-associated box (KRAB) domain of transcription regulators, allowing TRIM28 to suppress transcription from viral promoters, further aiding viral immune evasion (Rowe et al., 2010; Taka et al., 2022).

### 3.4 The influence of the PHD-BRD in virus infection

The PHD and BRD domains of TRIM proteins also play pivotal roles in regulating antiviral immunity. The PHD domain’s E3 ligase activity is essential for TRIM28’s self-SUMOylation, which inhibits immune gene expression mediated by IRF1, IRF3, and NF-κB during VSV infection. Full-length TRIM28, containing the PHD domain, suppresses chromatin accessibility of antiviral genes, while a truncated form lacking this domain does not, underscoring its importance in regulating antiviral immunity (Kuang et al., 2023). Conversely, the BRD of TRIM28 is critical for degrading the HIV-1 Tat protein, thereby repressing HIV-1 gene expression. TRIM28 interacts with Tat in microglial cells, facilitating its degradation via the proteasome pathway. Domain deletion studies reveal that while the RBCC domain is dispensable for Tat degradation, the BRD and, to a lesser extent, the PHD domain are essential for this process (Ait-Ammar et al., 2021). These findings highlight the multifaceted roles of TRIM domains in viral infections and their potential as targets for therapeutic intervention.

## 4 Post-translational modifications of C-VI TRIM proteins in viral infection

Despite their structural similarities, class VI TRIM proteins exhibit functional diversity during viral infections, due to post-translational modifications (PTMs), protein-protein interactions, subcellular localization, and domain-specific activities. PTMs, including SUMOylation, ubiquitination, and phosphorylation, are particularly influential, modulating TRIM protein stability, activity, and interactions, thereby shaping their roles in viral contexts.

### 4.1 SUMOylation

Although TRIM24 and TRIM33 SUMOylation have been shown to influence chromatin interaction (Appikonda et al., 2018) and TGFβ signaling (Fattet et al., 2013), respectively, their roles in viral infections remain underexplored. In contrast, TRIM28 SUMOylation has been extensively studied. It supports efficient virus replication in influenza A by inhibiting innate immune defences (Schmidt et al., 2019), maintains Epstein-Barr virus latency by repressing lytic replication (Bentz et al., 2015), and modulates adenoviral replication through chromatin decondensation (Bürck et al., 2016). Furthermore, SUMOylation enhances TRIM28’s recruitment to MMLV proviral DNA and represses proviral gene expression (Bin et al., 2015).

### 4.2 Ubiquitination

Ubiquitination also plays a key role in maintaining TRIM24 stability through its interaction with TRIM28, preventing its degradation and enhancing chromatin binding (Fong et al., 2018). Additionally, during VSV infection, TRIM28 ubiquitination by UBR5 inhibits its SUMOylation, enhancing antiviral responses (Yang et al., 2024).

### 4.3 Phosphorylation

Phosphorylation is a well-known modification among class VI TRIM proteins. For instance, the phosphorylation of TRIM24 at serine 768 (S768), mediated by ATM in response to DNA damage, leads to the destabilization and subsequent degradation of TRIM24 (Jain et al., 2014). Additionally, phosphorylation of TRIM24 at Ser1043 facilitates its translocation from the nucleus to the cytosol (Wei et al., 2022). However, during viral infections, phosphorylation is predominantly observed in TRIM28, with fewer reports in TRIM33. Notably, TRIM33 undergoes both phosphorylation and SUMOylation in response to EBV lytic infection (Cf et al., 2023).

Phosphorylation of TRIM28 plays a crucial role in regulating viral infections and their associated processes. For HIV, DNA-PK-mediated phosphorylation at S824 is essential for facilitating transcription by relieving paused RNA polymerase II at the HIV LTR, thus promoting viral replication (Zicari et al., 2020). During Merkel cell polyomavirus infection, phosphorylation at S824 induces cellular senescence and G2 arrest, counteracting viral genomic stress (Siebels et al., 2020). In adeno-associated virus infections, this modification inactivates TRIM28’s repression of the viral genome, aiding viral reactivation (Smith-Moore et al., 2018). Similarly, during human cytomegalovirus infection, mTOR-mediated phosphorylation suppresses TRIM28’s heterochromatin-inducing activity, facilitating viral reactivation (Rauwel et al., 2015). For highly pathogenic avian influenza virus infections, phosphorylation at S473 enhances TRIM28’s ability to induce cytokine production, boosting immune responses (Krischuns et al., 2018). Additionally, during Kaposi’s sarcoma-associated herpesvirus infection, MK2-mediated phosphorylation at S473 inactivates TRIM28, promoting STAT3 activation and inflammation (King, 2013). In EBV infections, phosphorylation sustains the expression of the viral lytic switch protein ZEBRA, facilitating reactivation and increased virus production (Li et al., 2017; Li et al., 2019). Lastly, in HSV-1 infections, TRIM28 phosphorylation relieves its repression on lytic gene transcription, modulating the balance between repression and activation (Tsai et al., 2022).

## 5 The TRIM24/TRIM28/TRIM33 complex in virus infection

The interaction among TRIM24, TRIM28, and TRIM33 forms a functionally significant complex that critically regulates viral infections and other cellular processes. Biochemical studies have demonstrated that these proteins co-purify and interact through their coiled-coil (CC) domains, which facilitate homo- and hetero-dimerization (Herquel et al., 2011; Randolph et al., 2022). For example, TRIM24 and TRIM28 frequently co-purify, with TRIM33 acting as a key interacting partner, and their interaction is essential for the repression of endogenous retroviruses (ERVs) in embryonic stem cells (Margalit et al., 2020). Structural studies using NMR and X-ray crystallography have revealed that the CC domains of these proteins mediate their dimerization, which is critical for their E3 ubiquitin ligase activity and chromatin-binding functions (Reymond, 2001; Stevens et al., 2019; Fiorentini et al., 2020). Additionally, the PHD-BRD cassette in TRIM24 and TRIM33 allows them to recognize specific histone modifications, such as H3K9me3 and acetylated lysines, which is essential for their role in chromatin remodeling and transcriptional regulation (Tsai et al., 2010; Bardhan et al., 2023). The functional significance of this complex is further highlighted by its role in antiviral defense. For instance, the TRIM24/TRIM28/TRIM33 complex suppresses Epstein-Barr virus (EBV) reactivation by repressing the lytic switch gene BZLF1. Disruption of this complex by EBV leads to the degradation of TRIM24 and modification of TRIM33, underscoring the importance of their interaction in maintaining viral latency (Cf et al., 2023). Moreover, the complex plays a role in tumor suppression, as simultaneous inactivation of TRIM24 and TRIM33 in mice leads to the development of hepatocellular carcinoma (HCC), highlighting their cooperative function in regulating cellular processes beyond viral infection (Herquel et al., 2011). These studies provide strong experimental evidence for the formation and functional significance of the TRIM24/TRIM28/TRIM33 complex, emphasizing its role in antiviral defense, chromatin regulation, and tumor suppression.

## 6 Small molecule ligands targeting C-VI TRIM protein domains in virus infection

The TRIM family of proteins, characterized by their diverse functional domains, play critical roles in cellular processes, with the RING domain being particularly significant due to its E3 ubiquitin ligase activity, which is essential for ubiquitination (Vunjak and Versteeg, 2019). While ubiquitination is a key function, certain TRIM proteins also exhibit ubiquitin-independent activities. Therapeutic strategies targeting ubiquitin signaling have been explored through the development of peptide-based inhibitors that bind to the proteasome, thereby preventing the degradation of ubiquitinated proteins (D’Amico et al., 2021). Proteasome inhibitors are widely used as research tools to study the ubiquitin-proteasome pathway (Hideshima and Anderson, 2012). Notably, proteasome-targeting therapeutics, including TRIM protein degraders, demonstrate considerable clinical promise; however, significant challenges remain. Their broad-spectrum effects on cellular processes can result in significant off-target effects and toxicity. For example, gastrointestinal toxicity, a frequent and dose-limiting adverse effect of proteasome inhibitors like bortezomib and carfilzomib, also persists despite newer formulations, warranting further mechanistic and translational investigation (Stansborough and Gibson, 2017). Additionally, limited tissue penetration and suboptimal bioavailability hinder their efficacy (Saraswat et al., 2023). Moreover, resistance mechanisms such as enhanced drug efflux can reduce the sustained effectiveness of these agents (Ming et al., 2023; Zhong et al., 2024). Overcoming these barriers will require refined molecular design, targeted delivery technologies, and comprehensive preclinical validation.

The intricate multi-domain architecture and adapter-like nature of TRIM proteins further complicate drug development efforts (D’Amico et al., 2021). A deeper understanding of how small molecule ligands interact with C-VI TRIM domains is essential for identifying druggable structural motifs. Bromodomain (BRD)-containing proteins have gained attention as a class of protein modules with therapeutic potential due to their ligand-binding capabilities (Ferri et al., 2016). Despite containing BRDs, TRIM proteins remain among the least studied targets in this category (Sekirnik et al., 2022). Recent advancements include the identification and optimization of N-benzyl-3,6-dimethylbenzo[d]isoxazol-5-amines as TRIM24 BRD inhibitors, with compounds 11d and 11h demonstrating potent inhibitory activity and selectivity in cancer cell proliferation assays (Hu et al., 2020). Notably, the TRIM24 BRD inhibitor IACS-9571 has shown promise as a latency-reversing agent for HIV-1, effectively reactivating proviral expression without inducing global T cell activation (Horvath et al., 2023). Additionally, the development of NP SUMOylation Interfering Peptides (NSIPs), particularly NSIP-III, has demonstrated efficacy in disrupting TRIM28-SARS2-NP interactions, inhibiting SARS2-NP SUMOylation, and impairing viral RNA binding (Ren et al., 2024).

More recently, dTRIM24, a selective degrader of the multidomain transcriptional regulator TRIM24, was shown to be more effective than the bromodomain inhibitor IACS-9571 in displacing TRIM24 from chromatin and modulating genome-wide transcription at its target genes (Gechijian et al., 2018). These findings not only establish TRIM24 as a transcriptional dependency in leukemia but also highlight the potential of domain-specific TRIM24-targeting molecules particularly degraders to precisely interrogate TRIM protein functions. Such approaches may be extended to study class VI TRIM proteins in the context of viral infection, where domain-targeting could modulate host-pathogen interactions or viral latency mechanisms.

Research into the histone H3 peptide binding profiles of TRIM24 and TRIM33 has provided valuable insights into their interactions with acetylated and methylated histone residues, laying the groundwork for the development of selective TRIM ligands (Sekirnik et al., 2022). These findings have implications for the design of proteolysis-targeting chimeras (PROTACs) and highlight the therapeutic potential of BRDs as ligandable protein modules. A classification system based on BRD binding site characteristics has been established to predict small molecule selectivity and refine inhibitor optimization strategies (Vidler et al., 2012).

## 7 Discussion

Class VI TRIM proteins (TRIM24, TRIM28, and TRIM33) are distinguished from other TRIM subfamilies by their unique C-terminal PHD-BRD cassette, which enables them to function as chromatin-associated transcriptional regulators (Sanchez and Zhou, 2011; Zaware and Zhou, 2019). While other TRIM subfamilies also contain the RBCC domain, Class VI TRIM proteins are particularly adept at recognizing specific histone modifications such as H3K9me3 and acetylated lysines as well as modulating chromatin accessibility during viral infections (Tsai et al., 2022; Bardhan et al., 2023). This ability to read the epigenetic code allows Class VI TRIM proteins to regulate gene expression in response to viral infection, a function that is less pronounced in other TRIM subfamilies. Consequently, Class VI TRIM proteins exert their antiviral or proviral effects through direct viral protein targeting, epigenetic regulation and chromatin remodelling, underscoring their unique contribution to antiviral defences.

Nonetheless, future research should leverage advanced proteomics and CRISPR-based technologies to further dissect the context-dependent roles of these proteins. For instance, mass spectrometry-based PTM mapping could identify novel modification sites on TRIM proteins, shedding light on how PTMs regulate their antiviral or proviral functions (Doll and Burlingame, 2015). Additionally, CRISPR-Cas9 could be employed to generate domain-specific knockouts or point mutations, enabling precise studies of how individual domains contribute to viral pathogenesis (Li et al., 2020). Furthermore, the formation and regulation of TRIM protein complexes, such as TRIM24/TRIM28/TRIM33, remain poorly understood. Proximity labelling techniques like BioID or APEX could map these complexes’ interactomes, revealing novel protein-protein interactions and their roles in viral replication (Guo et al., 2023). The development of selective small-molecule inhibitors targeting TRIM domains, particularly the RING and BRD domains, represents a promising therapeutic avenue. However, challenges such as off-target effects and toxicity must be addressed through structure-based drug design and virtual screening (Van Vleet et al., 2019). Finally, the role of TRIM proteins in emerging viral infections and their influence on host chromatin accessibility and gene expression warrants further exploration. Single-cell omics and *in vivo* models could provide insights into the physiological relevance of TRIM proteins in host defence, paving the way for novel antiviral therapies (Kirschenbaum et al., 2024). By addressing these gaps, future research can deepen our understanding of TRIM proteins’ roles in viral infections and inform the development of targeted antiviral strategies.

## References

[B1] Ait-Ammar A. Bellefroid M. Daouad F. Martinelli V. Assche J. Wallet C.. (2021) Inhibition of HIV-1 gene transcription by KAP1 in myeloid lineage. Scientific Reports 11 (1), 2692. doi: 10.1038/s41598-021-82164-w 33514850 PMC7846785

[B2] Ali H. Mano M. Braga L. Naseem A. Marini B. Vu D. M.. (2019). Cellular TRIM33 restrains HIV-1 infection by targeting viral integrase for proteasomal degradation. Nat. Commun. 10 (1), 926. doi: 10.1038/s41467-019-08810-0 30804369 PMC6389893

[B3] Anthony Massiah A. (2019). Zinc-binding B-box domains with RING folds serve critical roles in the protein ubiquitination pathways in plants and animals. Ubiquitin Proteasome System - Current Insights into Mechanism Cellular Regulation and Disease. 16 (1), 6. doi: 10.5772/intechopen.85895

[B4] Appikonda S. Thakkar K. N. Shah P. K. Dent S. Y. R. Andersen J. N. Barton M. C. (2018). Cross-talk between chromatin acetylation and SUMOylation of tripartite motif–containing protein 24 (TRIM24) impacts cell adhesion. J. Biol. Chem. 293, 7476–7485. doi: 10.1074/jbc.RA118.002233 29523690 PMC5950014

[B5] Bardhan I. Barman S. Roy A. Sudhamalla B. (2023). Novel insights into the recognition of acetylated histone H4 tail by the TRIM24 PHD-Bromo module. Biochem. J. 480, 629–647. doi: 10.1042/BCJ20230011 37075063

[B6] Bentz G. L. Moss C. R. Whitehurst C. B. Moody C. A. Pagano J. S. (2015). LMP1-induced sumoylation influences the maintenance of epstein-barr virus latency through KAP1. J. Virology. 89, 7465–7477. doi: 10.1128/JVI.00711-15 25948750 PMC4505653

[B7] Bin Chadi Hong Yu T. Hai Hao. (2015). Systematic identification of factors for provirus silencing in embryonic stem cells. Cell. 163, 230–245. doi: 10.1016/j.cell.2015.08.037 26365490 PMC4686136

[B8] Bürck C. Mund A. Berscheminski J. Kieweg L. Müncheberg S. Dobner T.. (2016). KAP1 is a host restriction factor that promotes human adenovirus E1B-55K SUMO modification. J. Virology. 90, 930–946. doi: 10.1128/JVI.01836-15 26537675 PMC4702688

[B9] Carthagena L. Bergamaschi A. Luna J. M. David A. Uchil P. D. Margottin-Goguet F.. (2009). Human TRIM gene expression in response to interferons. PloS One 4, e4894. doi: 10.1371/journal.pone.0004894 19290053 PMC2654144

[B10] Cf C.-H. Mh T. Uz S. S K. M E. Jf G.. (2023). Changes in SUMO-modified proteins in Epstein-Barr virus infection identifies reciprocal regulation of TRIM24/28/33 complexes and the lytic switch BZLF1. PloS Pathogens. 19, e1011477. doi: 10.1371/journal.ppat.1011477 37410772 PMC10353822

[B11] Chen Y. Y. Ran X. H. Ni R. Z. Mu D. (2023). TRIM28 negatively regulates the RLR signaling pathway by targeting MAVS for degradation via K48-linked polyubiquitination. J. Biol. Chem. 299, 104660. doi: 10.1016/j.jbc.2023.104660 37119745 PMC10165269

[B12] Cui Z. Zhou L. Zhao S. Li W. Li J. Chen J.. (2023). The host E3-ubiquitin ligase TRIM28 impedes viral protein GP4 ubiquitination and promotes PRRSV replication. Int. J. Mol. Sci. 24, 10965. doi: 10.3390/ijms241310965 37446143 PMC10341522

[B13] D’Amico F. Mukhopadhyay R. Ovaa H. Mulder M. P. C. (2021). Targeting TRIM proteins: A quest towards drugging an emerging protein class. ChemBioChem. 22, 2011–2031. doi: 10.1002/cbic.202000787 33482040 PMC8251876

[B14] Doll S. Burlingame A. L. (2015). Mass spectrometry-based detection and assignment of protein posttranslational modifications. ACS Chem. Biol. 10, 63–71. doi: 10.1021/cb500904b 25541750 PMC4301092

[B15] Fattet L. Ay A.-S. Bonneau B. Jallades L. Mikaelian I. Treilleux I.. (2013). TIF1γ requires sumoylation to exert its repressive activity on TGFβ signaling. J. Cell Science. 126, 3713–3723. doi: 10.1242/jcs.126748 23788427

[B16] Ferri E. Petosa C. McKenna C. E. (2016). Bromodomains: Structure, function and pharmacology of inhibition. Biochem. Pharmacol. 106, 1–18. doi: 10.1016/j.bcp.2015.12.005 26707800

[B17] Fiorentini F. Esposito D. Rittinger K. (2020). Does it take two to tango? RING domain self-association and activity in TRIM E3 ubiquitin ligases. Biochem. Soc. Trans. 48, 2615–2624. doi: 10.1042/BST20200383 33170204 PMC7752041

[B18] Fong K.-W. Zhao J. C. Song B. Zheng B. Yu J. (2018). TRIM28 protects TRIM24 from SPOP-mediated degradation and promotes prostate cancer progression. Nat. Communications. 9 (1), 5007. doi: 10.1038/s41467-018-07475-5 PMC6258673 30479348

[B19] Fonti G. Marcaida M. J. Bryan L. C. Träger S. Kalantzi A. S. Helleboid P. Y. J.. (2019). KAP1 is an antiparallel dimer with a functional asymmetry. Life Sci. Alliance. 2, e201900349. doi: 10.26508/lsa.201900349 31427381 PMC6701479

[B20] Gao Y. Xiang Y. H. Li C. Ye J. Lu Y. A. Ashraf U.. (2021). TRIM33 promotes spring viremia of carp virus replication by degrading the antiviral protein viperin_sv1. Aquaculture. 542, 736837. doi: 10.1016/j.aquaculture.2021.736837

[B21] Gechijian L. N. Buckley D. L. Lawlor M. A. Reyes J. M. Paulk J. Ott C. J.. (2018). Functional TRIM24 degrader via conjugation of ineffectual bromodomain and VHL ligands. Nat. Chem. Biol. 14, 405–412. doi: 10.1038/s41589-018-0010-y 29507391 PMC5866761

[B22] Giraldo M. I. Hage A. Tol S. Rajsbaum R. (2020). TRIM proteins in host defense and viral pathogenesis. Curr. Clin. Microbiol. Reports. 7, 101–114. doi: 10.1007/s40588-020-00150-8 PMC7414267 32837832

[B23] Guo J. Guo S. Lu S. Gong J. Wang L. Ding L.. (2023). The development of proximity labeling technology and its applications in mammals, plants, and microorganisms. Cell Communication Signaling 21 (1), 269. doi: 10.1186/s12964-023-01310-1 37777761 PMC10544124

[B24] Hage A. Rajsbaum R. (2019). To TRIM or not to TRIM: the balance of host–virus interactions mediated by the ubiquitin system. J. Gen. Virology. 100, 1641–1662. doi: 10.1099/jgv.0.001341 31661051 PMC7011758

[B25] Herquel B. Ouararhni K. Davidson I. (2011). The TIF1α-related TRIM cofactors couple chromatin modifications to transcriptional regulation, signaling and tumor suppression. Transcription. 2, 231–236. doi: 10.4161/trns.2.5.17725 22231120 PMC3265781

[B26] Hideshima T. Anderson K. C. (2012). Biologic impact of proteasome inhibition in multiple myeloma cells—From the aspects of preclinical studies. Semin. Hematology. 49, 223–227. doi: 10.1053/j.seminhematol.2012.04.006 PMC3383768 22726545

[B27] Horvath R. M. Brumme Z. L. Sadowski I. (2023). Inhibition of the TRIM24 bromodomain reactivates latent HIV-1. Sci. Reports. 13 (1), 556. doi: 10.1038/s41598-023-27765-3 PMC9832417 36631514

[B28] Hu Q. Wang C. Xiang Q. Wang R. Zhang C. Zhang M.. (2020). Discovery and optimization of novel N-benzyl-3,6-dimethylbenzo[d]isoxazol-5-amine derivatives as potent and selective TRIM24 bromodomain inhibitors with potential anti-cancer activities. Bioorganic Chem. 94, 103424. doi: 10.1016/j.bioorg.2019.103424 31776034

[B29] Hua F. Nass T. Parvatiyar K. (2024). TRIM28 facilitates type I interferon activation by targeting TBK1. Front. Immunol. 15. doi: 10.3389/fimmu.2024.1279920 PMC10940511 38495890

[B30] Jain A. K. Allton K. Duncan A. D. Barton M. C. (2014). TRIM24 Is a p53-Induced E3-Ubiquitin Ligase That Undergoes ATM-Mediated Phosphorylation and Autodegradation during DNA Damage. Mol. Cell. Biol. 34, 2695–2709. doi: 10.1128/MCB.01705-12 24820418 PMC4097665

[B31] King C. A. (2013). Kaposi’s sarcoma-associated herpesvirus kaposin B induces unique monophosphorylation of STAT3 at serine 727 and MK2-mediated inactivation of the STAT3 transcriptional repressor TRIM28. J. Virology. 87, 8779–8791. doi: 10.1128/JVI.02976-12 23740979 PMC3719813

[B32] Kirschenbaum D. Xie K. Ingelfinger F. Katzenelenbogen Y. Abadie K. Look T.. (2024). Time-resolved single-cell transcriptomics defines immune trajectories in glioblastoma. Cell. 187, 149–65.e23. doi: 10.1016/j.cell.2023.11.032 38134933

[B33] Krischuns T. Günl F. Henschel L. Binder M. Willemsen J. Schloer S.. (2018). Phosphorylation of TRIM28 enhances the expression of IFN-β and proinflammatory cytokines during HPAIV infection of human lung epithelial cells. Front. Immunol. 9. doi: 10.3389/fimmu.2018.02229 PMC6172303 30323812

[B34] Kuang M. Zhao Y. Yu H. Li S. Liu T. Chen L.. (2023). XAF1 promotes anti-RNA virus immune responses by regulating chromatin accessibility. Sci. Adv. 9 (33), eadg5211. doi: 10.1126/sciadv.adg5211 37595039 PMC10438455

[B35] Li X. Burton E. M. Bhaduri-Mcintosh S. (2017). Chloroquine triggers Epstein-Barr virus replication through phosphorylation of KAP1/TRIM28 in Burkitt lymphoma cells. PloS Pathogens. 13, e1006249. doi: 10.1371/journal.ppat.1006249 28249048 PMC5348047

[B36] Li X. Kozlov S. V. El-Guindy A. Bhaduri-Mcintosh S. (2019). Retrograde regulation by the viral protein kinase epigenetically sustains the epstein-barr virus latency-to-lytic switch to augment virus production. J. Virol. 93, e1006249. doi: 10.1128/JVI.00572-19 PMC6694827 31189703

[B37] Li X. Yan Z. Ma J. Li G. Liu X. Peng Z.. (2024). TRIM28 promotes porcine epidemic diarrhea virus replication by mitophagy-mediated inhibition of the JAK-STAT1 pathway. Int. J. Biol. Macromolecules. 254, 127722. doi: 10.1016/j.ijbiomac.2023.127722 37907173

[B38] Li H. Yang Y. Hong W. Huang M. Wu M. Zhao X. (2020). Applications of genome editing technology in the targeted therapy of human diseases: mechanisms, advances and prospects. Signal Transduction Targeted Ther. 5 (1), 1. doi: 10.1038/s41392-019-0089-y PMC6946647 32296011

[B39] Liang Q. Deng H. Li X. Wu X. Tang Q. Chang T. H.. (2011). Tripartite motif-containing protein 28 is a small ubiquitin-related modifier E3 ligase and negative regulator of IFN regulatory factor 7. J. Immunol. 187, 4754–4763. doi: 10.4049/jimmunol.1101704 21940674 PMC3197880

[B40] Margalit L. Strauss C. Tal A. Schlesinger S. (2020). Trim24 and trim33 play a role in epigenetic silencing of retroviruses in embryonic stem cells. Viruses. 12, 1015. doi: 10.3390/v12091015 32932986 PMC7551373

[B41] Mazurek S. Oleksiewicz U. Czerwińska P. Wróblewska J. Klimczak M. Wiznerowicz M. (2021). Disruption of RING and PHD domains of TRIM28 evokes differentiation in human iPSCs. Cells. 10, 1933. doi: 10.3390/cells10081933 34440702 PMC8394524

[B42] McAvera R. M. Crawford L. J. (2020). TIF1 proteins in genome stability and cancer. Cancers. 12, 2094. doi: 10.3390/cancers12082094 32731534 PMC7463590

[B43] Meulenberg J. J. Nieuwstadt A. P. Essen-Zandbergen A. Langeveld J. P. (1997). Posttranslational processing and identification of a neutralization domain of the GP4 protein encoded by ORF4 of Lelystad virus. J. Virology. 71, 6061–6067. doi: 10.1128/jvi.71.8.6061-6067.1997 9223499 PMC191865

[B44] Ming H. Li B. Jiang J. Qin S. Nice E. C. He W.. (2023). Protein degradation: expanding the toolbox to restrain cancer drug resistance. J. Hematol. Oncol. 16. doi: 10.1186/s13045-023-01398-5 PMC9872387 36694209

[B45] Ozato K. Shin D. M. Chang T. H. Morse H. C. (2008). TRIM family proteins and their emerging roles in innate immunity. Nat. Rev. Immunol. 8, 849–860. doi: 10.1038/nri2413 18836477 PMC3433745

[B46] Palmer W. S. Poncet-Montange G. Liu G. Petrocchi A. Reyna N. Subramanian G.. (2016). Structure-guided design of IACS-9571, a selective high-affinity dual TRIM24-BRPF1 bromodomain inhibitor. J. Medicinal Chem. 59, 1440–1454. doi: 10.1021/acs.jmedchem.5b00405 PMC4755933 26061247

[B47] Rajsbaum R. García-Sastre A. Versteeg G. A. (2014). TRIMmunity: the roles of the TRIM E3-ubiquitin ligase family in innate antiviral immunity. J. Mol. Biol. 426, 1265–1284. doi: 10.1016/j.jmb.2013.12.005 24333484 PMC3945521

[B48] Randolph K. Hyder U. D’Orso I. (2022). KAP1/TRIM28: transcriptional activator and/or repressor of viral and cellular programs? Front. Cell. Infection Microbiol. 12. doi: 10.3389/fcimb.2022.834636 PMC8904932 35281453

[B49] Rauwel B. Jang S. M. Cassano M. Kapopoulou A. Barde I. Trono D. (2015). Release of human cytomegalovirus from latency by a KAP1/TRIM28 phosphorylation switch. eLife. 4. doi: 10.7554/eLife.06068.035 PMC4384640 25846574

[B50] Ren J. Wang S. Zong Z. Pan T. Liu S. Mao W.. (2024). TRIM28-mediated nucleocapsid protein SUMOylation enhances SARS-CoV-2 virulence. Nat. Commun. 15 (1), 244. doi: 10.1038/s41467-023-44502-6 38172120 PMC10764958

[B51] Reymond A. (2001). The tripartite motif family identifies cell compartments. EMBO J. 20, 2140–2151. doi: 10.1093/emboj/20.9.2140 11331580 PMC125245

[B52] Rowe H. M. Jakobsson J. Mesnard D. Rougemont J. Reynard S. Aktas T.. (2010). KAP1 controls endogenous retroviruses in embryonic stem cells. Nature. 463, 237–240. doi: 10.1038/nature08674 20075919

[B53] Sanchez R. Zhou M. M. (2011). The PHD finger: a versatile epigenome reader. Trends Biochem. Sci. 36, 364–372. doi: 10.1016/j.tibs.2011.03.005 21514168 PMC3130114

[B54] Saraswat A. L. Vartak R. Hegazy R. Patel A. Patel K. (2023). Drug delivery challenges and formulation aspects of proteolysis targeting chimera (PROTACs). Drug Discov. Today 28, 103387. doi: 10.1016/j.drudis.2022.103387 36184017

[B55] Schmidt N. Domingues P. Golebiowski F. Patzina C. Tatham M. H. Hay R. T.. (2019). An influenza virus-triggered SUMO switch orchestrates co-opted endogenous retroviruses to stimulate host antiviral immunity. Proc. Natl. Acad. Sci. 116, 17399–17408. doi: 10.1073/pnas.1907031116 31391303 PMC6717285

[B56] Sekirnik A. R. Reynolds J. K. See L. Bluck J. P. Scorah A. R. Tallant C.. (2022). Identification of histone peptide binding specificity and small-molecule ligands for the TRIM33α and TRIM33β Bromodomains. ACS Chem. Biol. 17, 2753–2768. doi: 10.1021/acschembio.2c00266 36098557 PMC9594046

[B57] Shen Z. Wei L. Yu Z. B. Yao Z. Y. Cheng J. Wang Y. T.. (2021). The roles of TRIMs in antiviral innate immune signaling. Front. Cell. Infection Microbiol. 11. doi: 10.3389/fcimb.2021.628275 PMC8005608 33791238

[B58] Shibata M. Blauvelt K. E. Liem K. F. García-García M. J. (2011). TRIM28 is required by the mouse KRAB domain protein ZFP568 to control convergent extension and morphogenesis of extra-embryonic tissues. Development. 138, 5333–5343. doi: 10.1242/dev.072546 22110054 PMC3222210

[B59] Short K. M. Cox T. C. (2006). Subclassification of the RBCC/TRIM superfamily reveals a novel motif necessary for microtubule binding. J. Biol. Chem. 281, 8970–8980. doi: 10.1074/jbc.M512755200 16434393

[B60] Siebels S. Czech-Sioli M. Spohn M. Schmidt C. Theiss J. Indenbirken D.. (2020). Merkel cell polyomavirus DNA replication induces senescence in human dermal fibroblasts in a kap1/trim28-dependent manner. mBio 11 (2). doi: 10.1128/mBio.00142-20 PMC7064754 32156811

[B61] Smith-Moore S. Neil S. J. D. Fraefel C. Linden R. M. Bollen M. Rowe H. M.. (2018). Adeno-associated virus Rep proteins antagonize phosphatase PP1 to counteract KAP1 repression of the latent viral genome. Proc. Natl. Acad. Sci. 115, E3529–E3E38. doi: 10.1073/pnas.1721883115 29581310 PMC5899473

[B62] Stansborough R. L. Gibson R. J. (2017). Proteasome inhibitor-induced gastrointestinal toxicity. Curr. Opin. Supportive Palliative Care 11, 133–137. doi: 10.1097/SPC.0000000000000266 28333868

[B63] Stevens R. V. Esposito D. Rittinger K. (2019). Characterisation of class VI TRIM RING domains: linking RING activity to C-terminal domain identity. Life Sci. Alliance. 2, e201900295. doi: 10.26508/lsa.201900295 31028095 PMC6487577

[B64] Taka J. R. H. Sun Y. Goldstone D. C. (2022). Mapping the interaction between Trim28 and the &lt;scp<KRAB&lt;/scp< domain at the center of Trim28 silencing of endogenous retroviruses. Protein Science. 31 (10), e4436. doi: 10.1002/pro.4436 36173157 PMC9601868

[B65] Thiru A. Nietlispach D. Mott H. R. Okuwaki M. Lyon D. Nielsen P. R.. (2004). Structural basis of HP1/PXVXL motif peptide interactions and HP1 localisation to heterochromatin. EMBO J. 23, 489–499. doi: 10.1038/sj.emboj.7600088 14765118 PMC1271814

[B66] Tsai M. S. Chen S. H. Chang C. P. Hsiao Y. L. Wang L. C. (2022). Integrin-linked kinase reduces H3K9 trimethylation to enhance herpes simplex virus 1 replication. Front. Cell. Infection Microbiol. 12. doi: 10.3389/fcimb.2022.814307 PMC8957879 35350437

[B67] Tsai W. W. Wang Z. Yiu T. T. Akdemir K. C. Xia W. Winter S.. (2010). TRIM24 links a non-canonical histone signature to breast cancer. Nature. 468, 927–932. doi: 10.1038/nature09542 21164480 PMC3058826

[B68] van Gent M. Sparrer K. M. Gack M. U. (2018). TRIM proteins and their roles in antiviral host defenses. Annu. Rev. virology. 5, 385–405. doi: 10.1146/annurev-virology-092917-043323 29949725 PMC6186430

[B69] Van Vleet T. R. Liguori M. J. Lynch I. J. J. Rao M. Warder S. (2019). Screening strategies and methods for better off-target liability prediction and identification of small-molecule pharmaceuticals. SLAS Discovery. 24, 1–24. doi: 10.1177/2472555218799713 30196745

[B70] Varadi M. Bertoni D. Magana P. Paramval U. Pidruchna I. Radhakrishnan M.. (2024). AlphaFold Protein Structure Database in 2024: providing structure coverage for over 214 million protein sequences. Nucleic Acids Res. 52, 368–375. doi: 10.1093/nar/gkad1011 PMC10767828 37933859

[B71] Vidler L. R. Brown N. Knapp S. Hoelder S. (2012). Druggability analysis and structural classification of bromodomain acetyl-lysine binding sites. J. medicinal Chem. 55, 7346–7359. doi: 10.1021/jm300346w PMC3441041 22788793

[B72] Vunjak M. Versteeg G. A. (2019). TRIM proteins current biology 29, 42–44. doi: 10.1016/j.cub.2018.11.026 30668943

[B73] Walser R. Renshaw J. Milbradt A. G. (2016). Backbone resonance assignments for the PHD-Bromo dual-domain of the human chromatin reader TRIM24. Biomolecular NMR Assignments. 10, 207–211. doi: 10.1007/s12104-016-9668-9 26878853

[B74] Wei W. Chen Q. Liu M. Sheng Y. Ouyang Q. Feng W.. (2022). TRIM24 is an insulin-responsive regulator of P-bodies. Nat. Commun. 13 (1), 3972. doi: 10.1038/s41467-022-31735-0 35803934 PMC9270398

[B75] Wihandani D. M. Purwanta M. L. A. Mulyani W. R. W. Putra I. W. A. S. Supadmanaba I. G. P. (2023). New-onset diabetes in COVID-19: The molecular pathogenesis. BioMedicine. 13, 3–12. doi: 10.37796/2211-8039.1389 37168726 PMC10166251

[B76] Xi Q. Wang Z. Zaromytidou A. I. Xiang C.-T. L-F J. Kim H.. (2011). A poised chromatin platform for TGF-β Access to master regulators. Cell. 147, 1511–1524. doi: 10.1016/j.cell.2011.11.032 22196728 PMC3582033

[B77] Yang D. Geng T. Harrison A. G. Cahoon J. G. Xing J. Jiao B.. (2024). UBR5 promotes antiviral immunity by disengaging the transcriptional brake on RIG-I like receptors. Nat. Commun. 15 (1), 780. doi: 10.1038/s41467-024-45141-1 38278841 PMC10817939

[B78] Yuan P. Yan J. Wang S. Guo Y. Xi X. Han S.. (2021). Trim28 acts as restriction factor of prototype foamy virus replication by modulating H3K9me3 marks and destabilizing the viral transactivator Tas. Retrovirology 18, 1–18. doi: 10.1186/s12977-021-00584-y PMC8670036 34903241

[B79] Zaware N. Zhou M. M. (2019). Bromodomain biology and drug discovery. Nat. Struct. &amp; Mol. Biol. 26, 870–879. doi: 10.1038/s41594-019-0309-8 31582847 PMC6984398

[B80] Zhong G. Chang X. Xie W. Zhou X. (2024). Targeted protein degradation: advances in drug discovery and clinical practice. Signal Transduction Targeted Ther. 9 (1), 308. doi: 10.1038/s41392-024-02004-x PMC11539257 39500878

[B81] Zhu Q. Yu T. Gan S. Wang Y. Pei Y. Zhao Q.. (2020). TRIM24 facilitates antiviral immunity through mediating K63-linked TRAF3 ubiquitination. J. Exp. Med. 217 (7). doi: 10.1084/jem.20192083 PMC7336305 32324863

[B82] Zicari S. Sharma A. L. Sahu G. Dubrovsky L. Sun L. Yue H.. (2020). DNA dependent protein kinase (DNA-PK) enhances HIV transcription by promoting RNA polymerase II activity and recruitment of transcription machinery at HIV LTR. Oncotarget. 11, 699–726. doi: 10.18632/oncotarget.27487 32133046 PMC7041937

